# A Two-Gene-Based Diagnostic Signature for Ruptured Intracranial Aneurysms

**DOI:** 10.3389/fcvm.2021.671655

**Published:** 2021-08-13

**Authors:** Yuwang Li, Jie Qin

**Affiliations:** Department of Neurology, Tianjin Huanhu Hospital, Tianjin, China

**Keywords:** intracranial aneurysm, rupture, diagnosis, signature, immune analysis

## Abstract

**Background:** Ruptured intracranial aneurysm (IA) is a disease with high mortality. Despite the great progress in treating ruptured IA, methods for risk assessment of ruptured IA remain limited.

**Methods:** In this study, we aim to develop a robust diagnostic model for ruptured IA. Gene expression profiles in blood samples of 18 healthy persons and 43 ruptured IA patients were obtained from the Gene Expression Omnibus (GEO). Differential expression analysis was performed using limma Bioconductor package followed by functional enrichment analysis *via* clusterProfiler Bioconductor package. Immune cell compositions in ruptured IA and healthy samples were assessed through the CIBERSORT tool. Protein–protein interaction (PPI) was predicted based on the STRING database. Logistic regression model was used for the construction of predictive model for distinguishing ruptured IA and healthy samples. Real-time quantitative polymerase chain reaction (RT-qPCR) was performed to validate the gene expression between the ruptured IA and healthy samples.

**Results:** A total of 58 differentially expressed genes (DEGs) were obtained for ruptured IA patients compared with healthy controls. Functional enrichment analysis showed that the DEGs were enriched in biological processes related to neutrophil activation, neutrophil degranulation, and cytokine–cytokine receptor interaction. Notably, immune analysis results proved that the rupture of IA might be related to immune cell distribution. We further identified 24 key genes as hub genes using the PPI networks. The logistic regression model trained based on the 24 key genes ultimately retained two genes, i.e., IL2RB and CCR7, which had great potential for risk assessment for rupture of IA. The RT-qPCR further validated that compared with the healthy samples, the expression levels of IL2RB and CCR7 were decreased in ruptured IA samples.

**Conclusions:** This study might be helpful for cohorts who have a high risk of ruptured IA for early diagnosis and prevention of the disease.

## Introduction

An intracranial aneurysm (IA) is traditionally defined as an abnormal protrusion that appears on the walls of intracranial arteries and is associated with high morbidity and mortality (1). Rupture of IAs leads to subarachnoid hemorrhage (SAH), which may lead to severe consequences. Although ~2–5% of the population develops IAs during life, most IAs do not rupture (2, 3). SAH has a fatality rate of ~50%, with an incidence rate of ~9 per 1,000,000 person-years (4). Therefore, identifying IAs with a high risk of rupture and providing timely intervention may be imperative for improving the risk assessment and treatment.

Previous studies on factors that contribute to an increased risk of rupture of IAs have mainly focused on morphological factors and primary diseases (5, 6). Recently, hemodynamic factors, such as the oscillatory shear index, wall shear stress, and blood flow velocity, have been shown to be associated with the formation, progression, and rupture of IAs (7, 8). Wall shear stress is regarded as the most important factor for IA initiation and rupture. Nevertheless, the presence of wall shear stress cannot be detected in clinical practice. Conversely, geometric features can be used to identify aneurysms that are at risk of rupture. The relationship between morphological factors and the rupture rate of IAs has become a research hot spot. Although many studies have evaluated the relationship between geometric features and IA rupture, there are still great differences (8–10). In addition, genetic factors have been shown to be closely associated with ruptured IAs, and significant efforts have sought to identify genetic biomarkers for IA rupture (11). To further identify the potential genes related to the pathogenesis of ruptured IAs, which may provide novel molecular signatures, we performed this study and focused on the molecular mechanisms of IA progression and rupture to identify molecules that affect IA rupture. Furthermore, we constructed a reliable logistic regression model to assess the risk of a ruptured IA, which might be beneficial for the timely diagnosis and subsequent treatment of the disease.

Here, we investigated the potential of gene expression as a diagnostic marker for ruptured IAs. We finally identified interleukin (IL) 2RB and CCR7 as diagnostic genes for ruptured IAs, and a logistic regression model constructed by these two genes was found to efficiently distinguish patients with ruptured IAs from unruptured IA samples as well as from healthy control samples.

## Materials and Methods

### Gene Expression Dataset

The mRNA expression profile dataset GSE36791 was downloaded from the Gene Expression Omnibus (GEO, https://ncbi.nlm.nih.gov/geo). There were 18 blood samples from healthy people and 43 blood samples from patients with ruptured IAs in the GSE36791 dataset. Whole-genome mRNA levels of those samples were detected using an Illumina HumanHT-12 V4.0 expression beadchip. Among the 43 disease samples, there were 21 female samples and 22 male samples (Supplementary Figure 1), and there was no significant difference (chi-square test, χ^2^ = 0.023256, *P*-value = 0.8788). The clinical characteristics of the samples are shown in Supplementary Table 1 (12). The mRNA expression profile of dataset GSE6551 was also obtained from the GEO database and included eight ruptured IA samples and six unruptured IA samples.

### Differential Expression Analysis

The GSE36791 dataset was first normalized using the robust multi-array average (RMA) method and standardized by logarithmic transformation. The Limma Bioconductor package in R was used for differential expression analysis based on the normalized mRNA levels, and the genes that met the threshold of an absolute value of log2-based fold change (|log2FC|) >1 and a false discovery rate (FDR) ≤ 0.05 were defined as differentially expressed genes (DEGs).

### Functional Enrichment Analysis

Gene Ontology (GO, http://geneontology.org) terms (including biological process, molecular function, and cellular component) and Kyoto Encyclopedia of Genes and Genomes (KEGG, https://www.genome.jp/kegg/) pathway enrichment analyses were performed using the “*ClusterProfiler*” Bioconductor package. A *P*-value <0.05 was considered statistically significant.

### Immune Cell Analysis

We used the CICERSORT R package to characterize the composition of immune cells for ruptured IAs and normal samples based on their gene expression profiles (13). The relative proportions of a total of 22 immune cells in each sample were calculated.

### Protein–Protein Interaction Network

The interactions among the proteins encoded by the DEGs were predicted using the STRING database (https://string-db.org/cgi/input.pl) (14) with the criteria of a minimum required interaction score >0.4. The protein–protein interaction (PPI) network was visualized using the Cytoscape software (15). A modular analysis of the PPI network was performed to identify densely interacting genes using the MCODE plug-in for the Cytoscape software.

### Construction of a Logistic Regression Model

Multivariate logistic regression analysis was performed using the glm R package. Sample types including ruptured IA samples and healthy samples were used as categorical responsive values, and gene expression values were used as continuous predictive variables. Then, stepwise regression was performed to further identify pivotal genes to simplify the model. Receiver operating characteristic (ROC) curve analysis was used to evaluate the model to distinguish ruptured IA and normal samples.

### Patients

The patients (≥18 years old, *n* = 15) who were diagnosed with ruptured IA in Tianjin Huanhu Hospital were selected for this research. The patients with other diseases or complications were excluded. A total of 15 healthy persons over 18 years old were also recruited. Written informed consents were obtained from all participants, and the research was approved by the ethics committee of our hospital (2021-048).

### RNA Extraction and Real-Time Quantitative Polymerase Chain Reaction

Venous whole blood of the 30 samples was collected, and total RNA was extracted by using a Blood RNA extraction kit (Tiangen, Beijing, China, cat#DP433). After determination of the RNA concentration and purity, reverse transcription was performed with a reverse transcription reagent (Qiagen, Hilden, Germany, cat#205111). For each sample, the amount of template RNA was the same. Real-time quantitative polymerase chain reaction (RT-qPCR) was conducted using a RT-qPCR kit (Solarbio, cat#SR1110, Beijing, China) on an ABI 7500 Real-Time PCR System (Thermo Fisher, Waltham, MA, USA). GAPDH was used as the internal control gene. The primer sequences for RT-qPCR are shown in Table 1, and the conditions were as follows: 95°C for 10 min, followed by 40 cycles consisting of 95°C for 10 s, 60°C for 20 s, and 72°C for 30 s. The results were calculated by the 2^−ΔΔct^ method.

**Table 1:** Primer sequences for RT-PCR.

**Genes**	**Forward primer (5′-3′)**	**Reverse primer (5′-3′)**	**Product length (bp)**	**Tm (°C)**
IL2RB	GCTGATCAACTGCAGGAACAC	GCGAAGAGAGCCACTTCTGG	131	60
CCR7	AACCCCTCCCTCCATCGTTT	CTTTGATCACGCGGAGGCA	176	61
GAPDH	GCAAATTCCATGGCACCGTC	AGCATCGCCCCACTTGATTT	110	59

## Results

### DEGs

**Figure 1:**
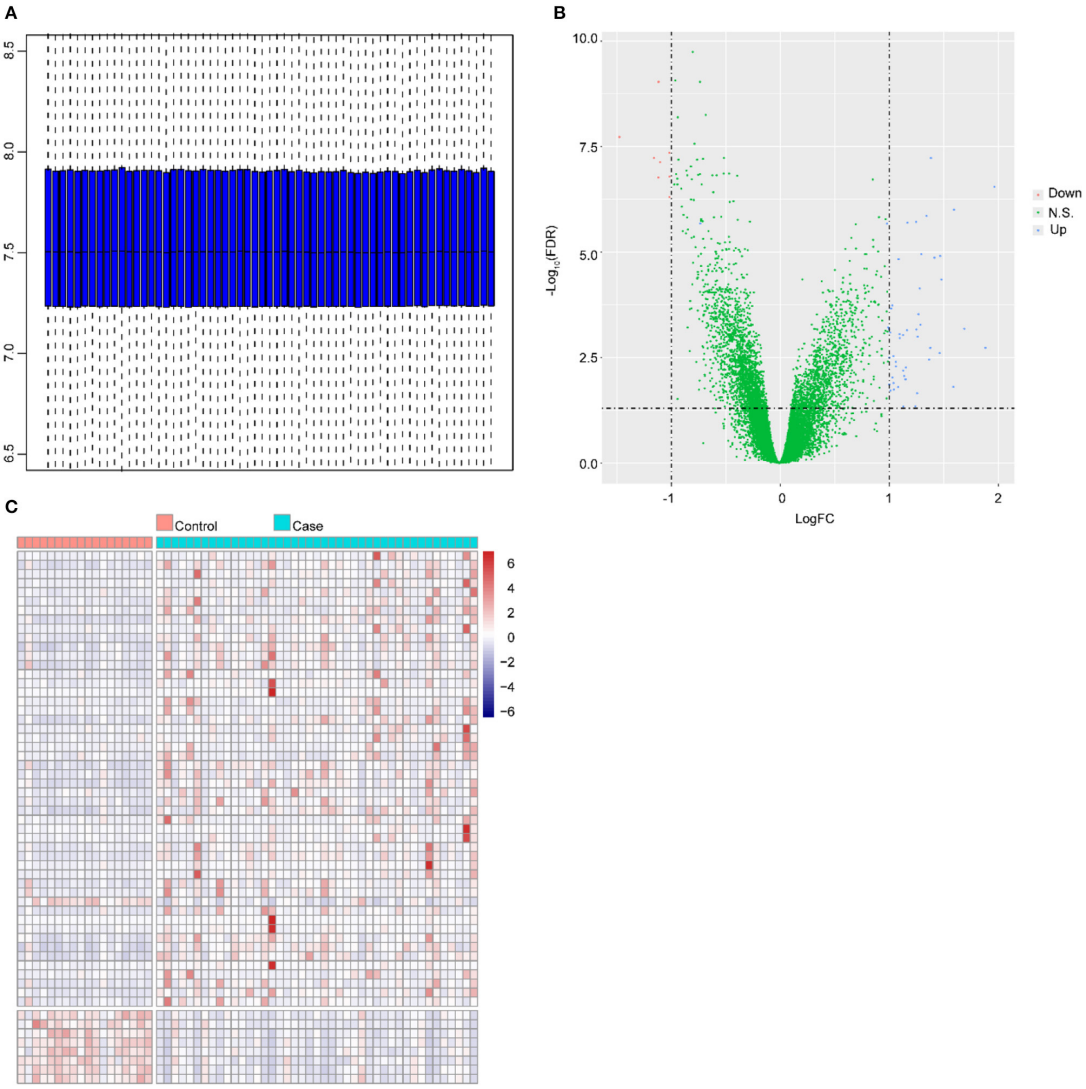
Differential expression analysis. **(A)** Distribution of expression in all samples after normalization. The horizontal axis and vertical axis represent samples and gene expressions, respectively. The line in the middle of the blue region represents the median value. The upper edge of the blue region represents the upper quartile, and the bottom edge of the blue region represents the lower quartile. **(B)** Volcano plot of DEGs. The *X*-axis and *Y*-axis represent log2FC and –log10 (FDR), respectively. The blue dots and red dots represent upregulated and downregulated genes, respectively. **(C)** Heat map of expression of DEGs in all samples. The horizontal axis and vertical axis represent the sample type and genes, respectively. Red: high expression, blue: low expression.

The normalized mRNA levels across all samples are shown in Figure 1A; these findings indicated homogeneous mRNA expression among all samples that was suitable for further analysis. A total of 58 DEGs were identified in the ruptured IA group compared with the healthy control group, namely, 50 upregulated genes and 8 downregulated genes. The distribution of log2FC and adjusted *P*-value are illustrated in Figure 1B. Figure 1C shows the expression of the 58 DEGs stratified by the changed direction of expression and sample group.

### Functional Enrichment Analysis of DEGs

**Figure 2:**
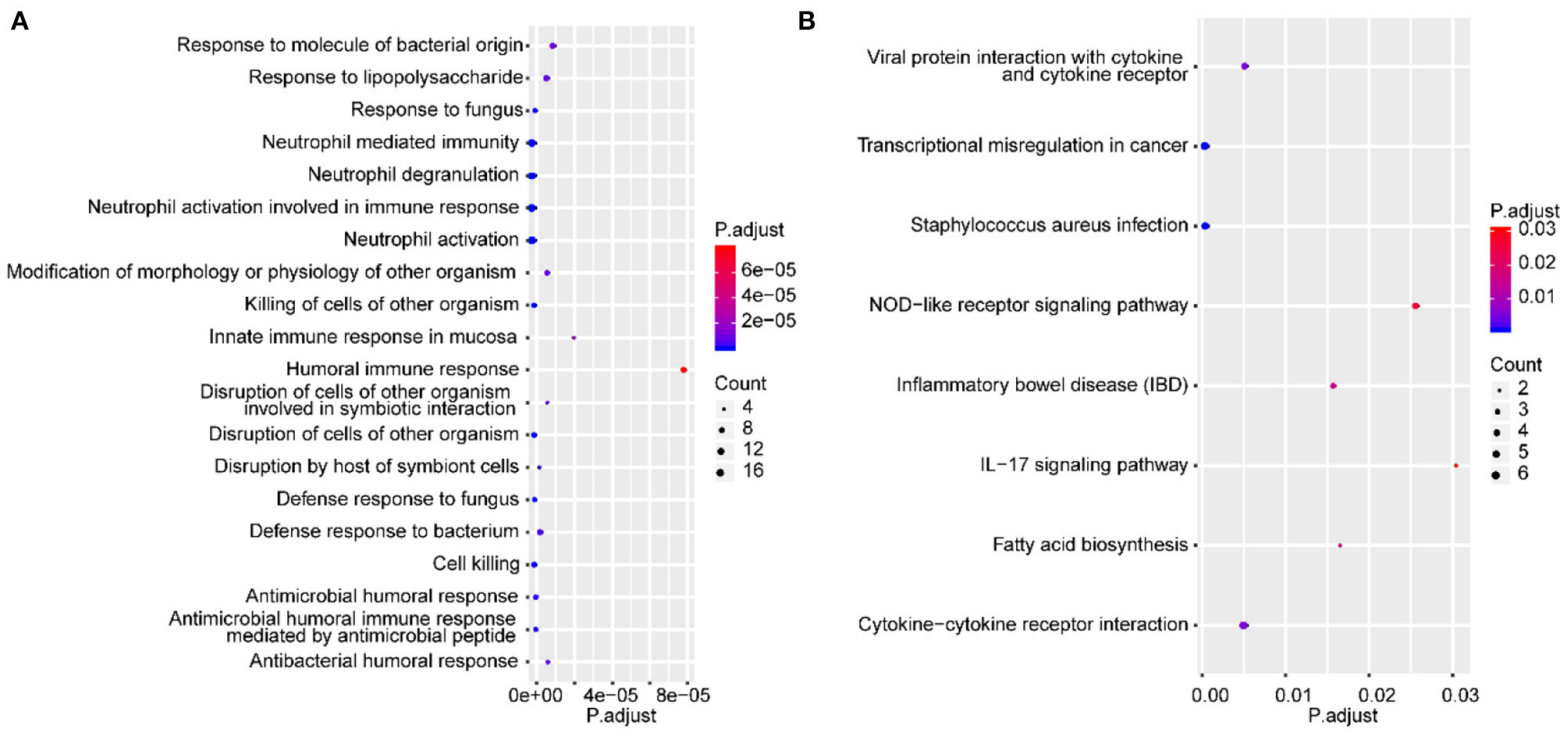
Functional enrichment analysis of DEGs. The top 20 most significantly enriched GO terms **(A)** and the full list of significantly enriched KEGG pathways based on DEGs **(B)**. The *X*-axis and *Y*-axis represent the adjusted *P*-value and biological process or pathway, respectively.

The GO terms and KEGG pathway enrichment analysis of DEGs are presented in Supplementary Tables 2, 3, respectively. In addition, the top 20 most significantly enriched GO terms and a full list of significantly enriched KEGG pathways are shown in Figures 2A,B, respectively. These findings illustrated that 58 DEGs were significantly enriched in biological processes (BPs) related to the immune response, such as neutrophil activation and neutrophil degranulation. Furthermore, KEGG pathway analysis revealed that these 58 genes were mainly enriched in inflammatory and immune responses and cancer-related pathways. Immune cells have been reported to be closely related to the occurrence and development of IAs (16). Notably, Miyata et al. demonstrated that there was considerable immune cell infiltration, such as macrophage infiltration in IA lesions (17). Combining the enrichment results of this study, we speculated that immune cells might be related to the rupture of IAs.

### Immune Cells May Be Associated With Ruptured IAs

**Figure 3:**
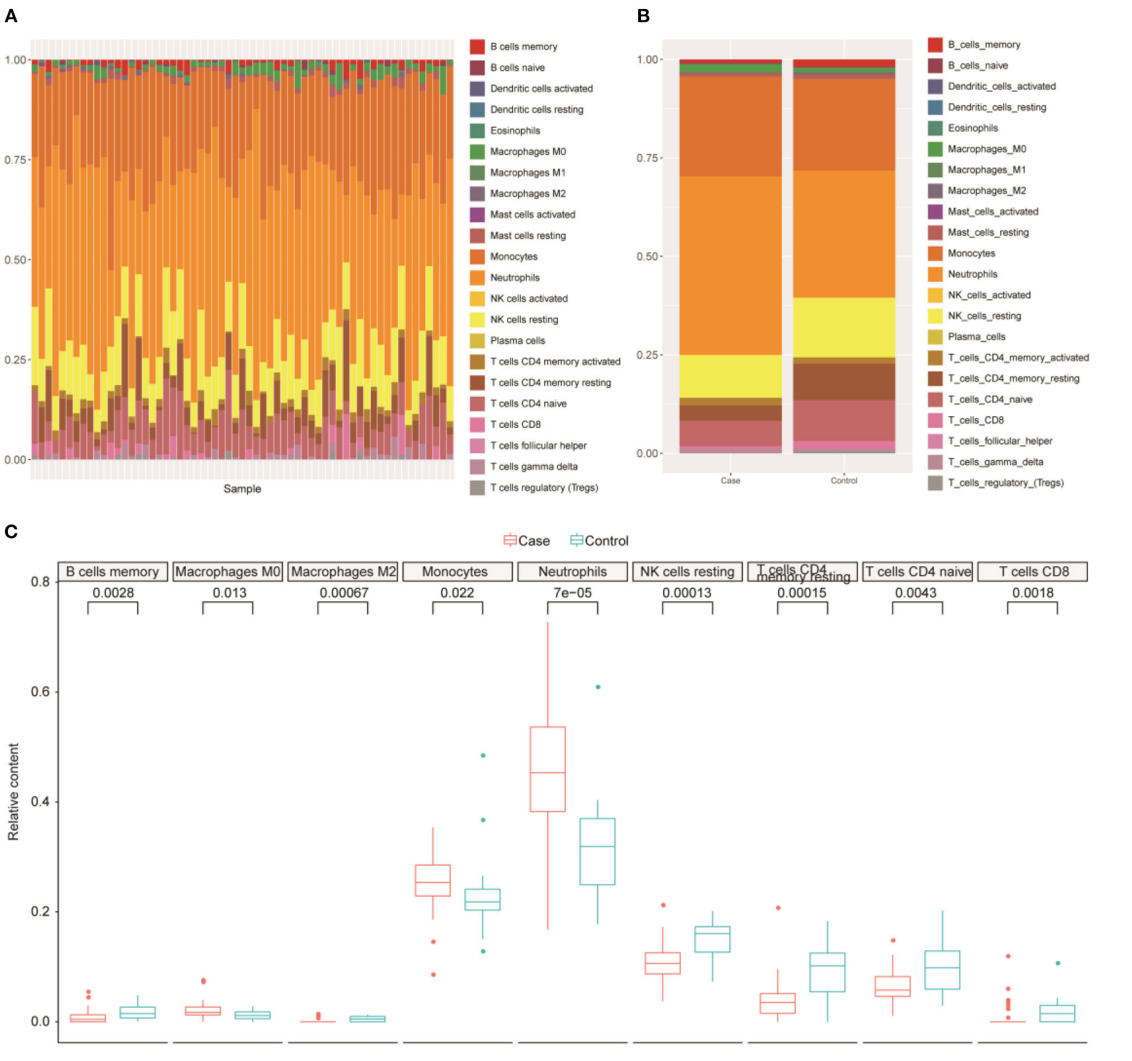
Immune cell analysis. **(A)** Differences of 22 immune cells among all samples. **(B)** Distribution of immune cells for samples between normal and intracranial aneurysm groups. *X*-axis, samples type. *Y*-axis, the proportion of immune cells. **(C)** Nine immune cell types with significant difference in relative proportions between normal and intracranial aneurysm groups.

The GO and KEGG results illustrated that DEGs were significantly enriched in several BPs or pathways related to immune or inflammatory responses; thus, we further analyzed the immune cell composition in different samples. Using the CIBERSORT tool, we analyzed the differences in 22 immune cells between the healthy control group and the ruptured IA group (Figure 3A). The results indicated that immune cells including B_cells_memory, T_cells_CD8, T_cells_CD4_memory_resting, T_cells_CD4_naive, Macrophages_M0, Macrophages_M2, NK_cells_resting, monocytes, and neutrophils were significantly different between ruptured IA and normal samples (*P* < 0.05, Wilcoxon test) (Figures 3B,C). In summary, the above results suggested that immune cells are closely associated with ruptured IAs.

### Potential Core Genes in Ruptured IAs

**Figure 4:**
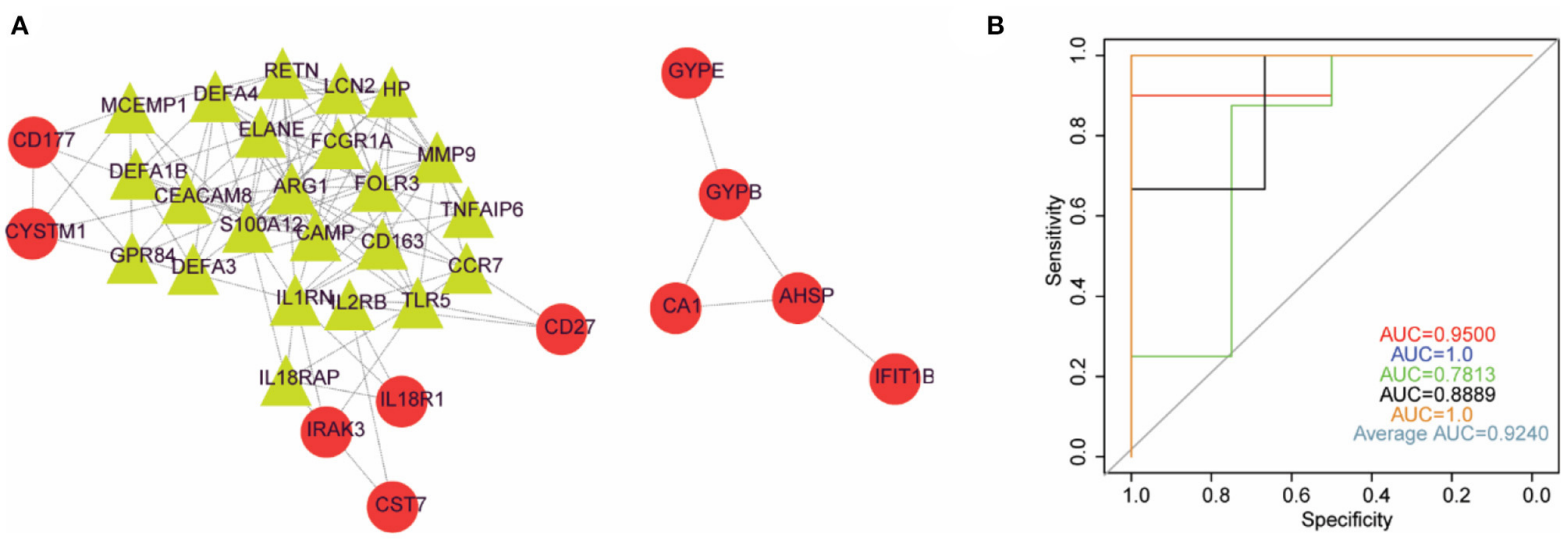
Logistic regression model could basically differentiate intracranial aneurysms from normal samples. **(A)** PPI network diagram constructed by DEGs. **(B)** The ROC curve of 5-fold cross-validation of the model constructed by IL2RB and CCR7. *X*-axis: false positive rate (FPR). *Y*-axis: true positive rate (TPR).

A PPI network was constructed based on the 58 DEGs and included 35 nodes and 117 edges (Figure 4A). Then, a subnetwork composed of 24 nodes and 102 edges was obtained using the MCODE plug-in for modular analysis (Figure 4A). Those 24 DEGs were considered potential critical genes in ruptured IAs.

### Two-Gene-Based Logistic Regression Model

We applied the stepwise regression method to the 24 DEGs contained in the subnetwork using their expression values and sample type (ruptured IA vs. normal) as continuous predictive variables and categorical responsive values, respectively, and finally retained six genes, including MMP9, RETN, LCN2, CAMP, CCR7, and IL2RB. The *P*-values of IL2RB and CCR7 were <0.05 in the logistic regression model, implying that IL2RB and CCR7 might have a greater contribution to the model than the other genes. Furthermore, the expression levels of IL2RB and CCR7 were decreased in ruptured IA samples compared with healthy control samples. Therefore, we finally constructed a logistic regression model based on IL2RB and CCR7. The performance of this model in distinguishing ruptured IA and normal samples was evaluated by 5-fold cross-validation. The area under the curve (AUC) value was used to assess the accuracy of the models in the 5-fold cross-validation. As a result, the AUC ranged from 0.78 to 1 with an average AUC of 0.92, indicating the reliability of this model in differentiating ruptured IA from normal samples (Figure 4B).

Moreover, the performance of this established logistic regression model based on IL2RB and CCR7 genes was assessed in another dataset, GSE6551, which included eight ruptured IA samples and six unruptured IA samples. As shown in Supplementary Figure 2, the AUC value for dataset GSE6551 was 0.81, indicating that the logistic regression model established with IL2RB and CCR7 in the GSE36791 dataset could also differentiate ruptured IA samples from the unruptured IA samples in dataset GSE6551.

### Validation of the IL2RB and CCR7 Expressions in Ruptured IA Patients and Healthy Controls

**Figure 5:**
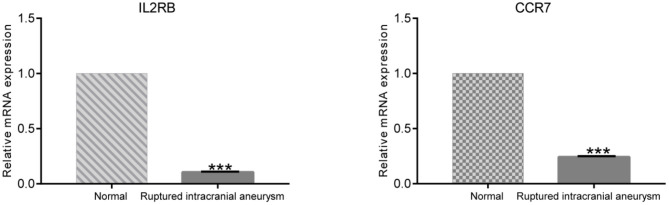
The result of RT-qPCR illustrating the mRNA expression levels of IL2RB and CCR7 in ruptured IA patients (*n* = 15) and healthy controls (*n* = 15). ^***^P < 0.001.

To further validate the differential expressions of IL2RB and CCR7 between ruptured IA patients and healthy controls, the RT-qPCR was conducted. As shown in Figure 5, the expression levels of IL2RB and CCR7 in ruptured IA samples were significantly lower than those in healthy controls (*P* < 0.001), which is consistent with the bioinformatic analysis.

## Discussion

IA has become a common disease with ~1–2% incidence worldwide (18). Unruptured IAs are increasingly being detected as cross-sectional imaging techniques are used more frequently in clinical practice (19). Recently, a series of key genes in plasma have been predicted to be potential biomarkers for the diagnosis of or prognostication for IAs. For instance, Li et al. indicate that several miRNAs in serum may be involved in the regulation of IAs, such as miRNA-21, miRNA-22, and miRNA-720; these are considered promising biomarkers for the early diagnosis of ruptured IAs (20). Tutino et al. established a diagnostic model with 18 genes based on whole blood transcriptomes that showed high accuracy in predicting unruptured IAs (21). Marchese et al. screened for several markers in peripheral blood of patients with cerebral aneurysms through transcriptional analysis (22). Circulating neutrophils were shown to carry a signature related to IAs, providing a possibility to diagnose patients with IAs based on biomarkers in peripheral blood (23). Based on the circulating neutrophil transcriptome data, Meng et al. constructed a model to predict unruptured IAs (24); this model was further improved by the integration of machine learning approaches and large cohorts (25). Quan et al. reported that the expression of F2-isoprostanes and F4-neuroprostanes in the plasma of patients with IAs is significantly upregulated compared with healthy subjects, and these may be considered potential biomarkers for IA development (26). Kao et al. identified that patients with high levels of aneurysmal cyclophilin A (CyPA) in plasma were 15.66 times more likely to have worse neurological outcomes than those with low levels; this may be a significant prognostic biomarker for poor neurological outcome in IA (27). Compared with serum or tissue samples, blood samples are readily available. In this study, we identified 58 DEGs, namely, 50 upregulated and 8 downregulated genes in blood samples from patients with ruptured IAs compared with healthy subjects, suggesting that these DEGs are potentially involved in ruptured IAs.

The formation of an IA is initiated by hemodynamically triggered endothelial cell dysfunction followed by an inflammatory reaction (28). Previous studies demonstrated that inflammation is one of the factors that participates in the formation, progression, and rupture of IAs (29). To determine the reliability of our analysis, GO and KEGG pathway enrichment analyses based on the 58 DEGs were performed. As expected, these DEGs were significantly and majorly enriched in the BPs involved in inflammatory and immune responses. Indeed, the dysfunction of endothelial cells, smooth muscle cells, macrophages, and lymphocytes, as well as their secreted cytokines, collectively contribute to the formation, growth, and rupture of IAs (30). Therefore, the differences in 22 immune cells were analyzed, and nine immune cells were found to exhibit significantly different proportions in patients with ruptured IAs compared with healthy subjects. These results confirm that inflammatory and immune responses are closely associated with the rupture of IAs and are consistent with the previous research results including downregulation of transcripts related to positive leukocyte activation, T-cell differentiation, and CD4+ and CD8+ lymphocytes in IA rupture (12, 31).

PPI networks are viable tools to elucidate cell functions, disease machinery, and drug design/repositioning (32). Here, PPI analysis was performed using the 58 DEGs, and 24 DEGs were screened based on the MCODE method; these were considered key genes involved in the rupture of IAs. To construct a strong explanatory model with as few genes as possible, a stepwise regression analysis was performed. Finally, two optimal genes, namely, IL2RB and CCR7, which were predicted to be closely associated with IA development and rupture, were selected. The RT-qPCR results validated the differential expression of IL2RB and CCR7 between ruptured IA and healthy control samples. IL-2/IL-2R is a cytokine of the chemokine family, and its coding gene mainly contains two different subunits: IL2RA and IL2RB (33, 34). IL-2 can enhance the proliferative ability of Treg cells in patients with IA, which is beneficial for inhibition of inflammation and tissue protection; moreover, compared with the healthy controls, the IL-2 abundance was relatively decreased in patients with IA (35). IL2RA, also known as CD25 (36), has been implicated in neurological pathology. In a model of stroke, neurogenesis was found to be repressed after the removal of ILR2A-T cells (37). The allele (rs2104286 G) of IL2RA was also found to have a higher frequency in patients with neuromyelitis optica than in healthy controls, suggesting that this IL2RA allele is associated with an increased risk of this neurological disorder (37). L2RB polymorphisms are also associated with lung cancer risk in the Chinese Han population (38). In addition, a series of studies have demonstrated that IL2RA and IL2RB participate in inflammatory processes in various human autoimmune diseases such as type I diabetes (39), rheumatoid arthritis (RA) (40), and multiple sclerosis (MS) (41). The chemokine receptor CCR7 is a member of the chemokine receptor superfamily and controls a diverse array of migratory events in adaptive immunity following antigen encounter by immunocytes (42). Although there is no direct evidence that CCR7 is associated with IA, the expression of CCR7 is significantly downregulated in abdominal aortic aneurysm (43). The migration of mature dendritic cells (DCs) into the draining lymph node (dLN) is thought to depend solely on CCR7 (44). CCR7 upregulation also induces prostate cancer cell migration (45). Finally, a logistic regression model was constructed based on IL2RB and CCR7. The 5-fold cross-validation method and AUC value of the ROC curve all suggested that the logistic regression model could reliably separate patients with ruptured IAs from healthy subjects. Furthermore, the performance of this logistic regression model based on IL2RB and CCR7 was assessed in another dataset, indicating that this model could also differentiate ruptured IA samples from unruptured IA samples. Therefore, our model could differentiate ruptured IA samples from unruptured IA samples as well as from healthy control samples. Due to the fact that ruptured IA causes SAH, which leads to high mortality (46), our predictive model can potentially be applied to risk assessment for cohorts with a high risk of ruptured IA for early prevention and further diagnosis of this disease.

However, there are still some limitations in our study. (1) Patients with ruptured IAs and healthy controls had different smoking and drinking statuses, which may have led to the difference in gene expressions. (2) The sample size in dataset GSE6551, which was used to evaluate the performance of the model in distinguishing ruptured and unruptured IA samples, was small, and a validation cohort with a larger sample size would be helpful for further assessment of the model. (3) Although our results indicated that the DEGs IL2RB and CCR7 between ruptured IA and healthy control samples may be closely related to IA rupture, it remains unclear whether the differences in gene expression are the result or the cause of ruptured IAs. In either case, the identification of IL2RB and CCR7 could provide insight into the underlying biology; however, further research is needed to better explain the detailed mechanism.

## Conclusion

In summary, our study identified 58 DEGs that are potentially involved in IA rupture. A reliable logistic regression model based on two optimal risk genes, namely, IL2RB and CCR7, was constructed and may have certain practical value, suggesting that IL2RB and CCR7 may be considered potential rapid diagnostic signatures for ruptured IAs.

## Data Availability Statement

The datasets generated during the current study are available in the (GSE36791) and (GSE6551) repositories, (GEO, https://ncbi.nlm.nih.gov/geo).

## Ethics Statement

The studies involving human participants were reviewed and approved by Tianjin Huanhu Hospital Ethics Committee. The patients/participants provided their written informed consent to participate in this study.

## Author Contributions

All authors listed have made a substantial, direct and intellectual contribution to the work, and approved it for publication.

## Conflict of Interest

The authors declare that the research was conducted in the absence of any commercial or financial relationships that could be construed as a potential conflict of interest.

## Publisher's Note

All claims expressed in this article are solely those of the authors and do not necessarily represent those of their affiliated organizations, or those of the publisher, the editors and the reviewers. Any product that may be evaluated in this article, or claim that may be made by its manufacturer, is not guaranteed or endorsed by the publisher.

## Supplementary Material

Supplementary Figure 1 The details of samples.

Supplementary Figure 2 The ROC curve. X-axis: false positive rate; Y-axis: true positive rate. The performance of the model was evaluated by the AUC value, and a larger AUC value within the range from 0 to 1 reflected a superior performance.

Supplementary Table 1 Clinical characteristics of the samples.

Supplementary Table 2 Full list of significantly enriched GO terms of DEGs.

Supplementary Table 3 Full list of KEGG pathways of DEGs.
