# Supplementary figures and images for: A Two-Gene-Based Diagnostic Signature for Ruptured Intracranial Aneurysms
Source: Front Cardiovasc Med. 2021 Aug 13;8:671655. doi: 10.3389/fcvm.2021.671655 (PMC8414364; doi:10.3389/fcvm.2021.671655)

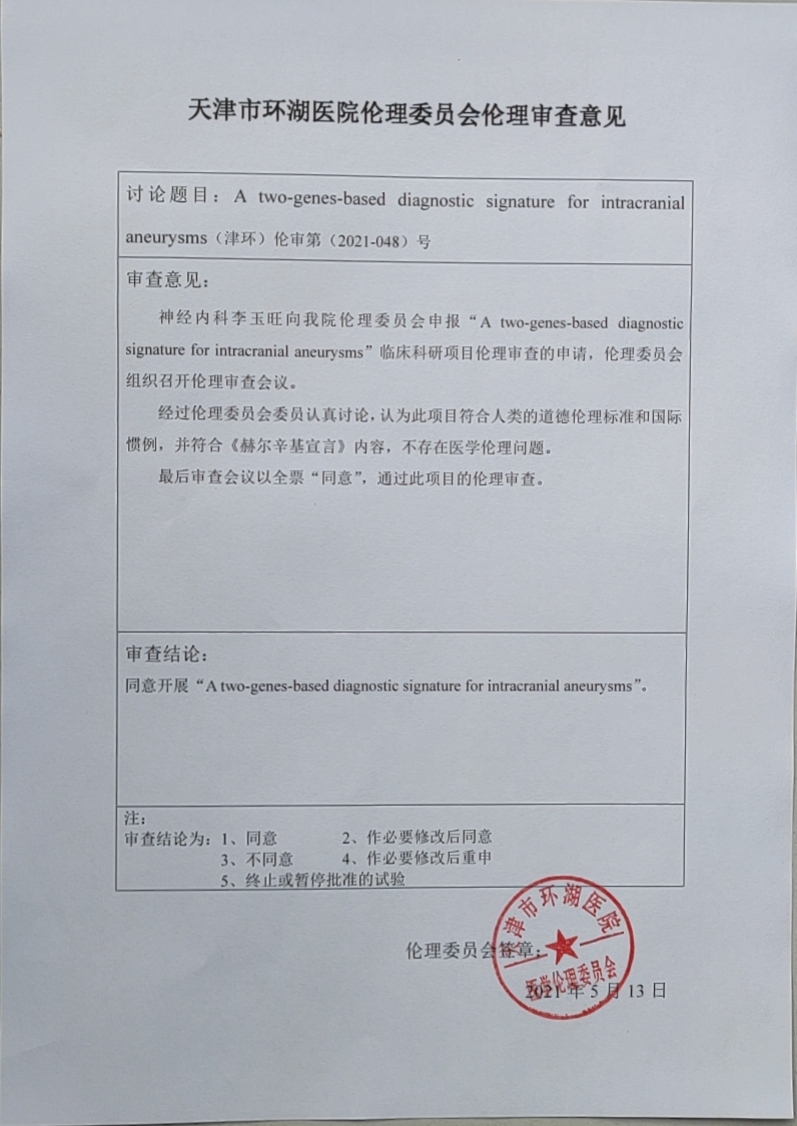

Supplement: Supplementary file 1 [file Image_1.JPEG]

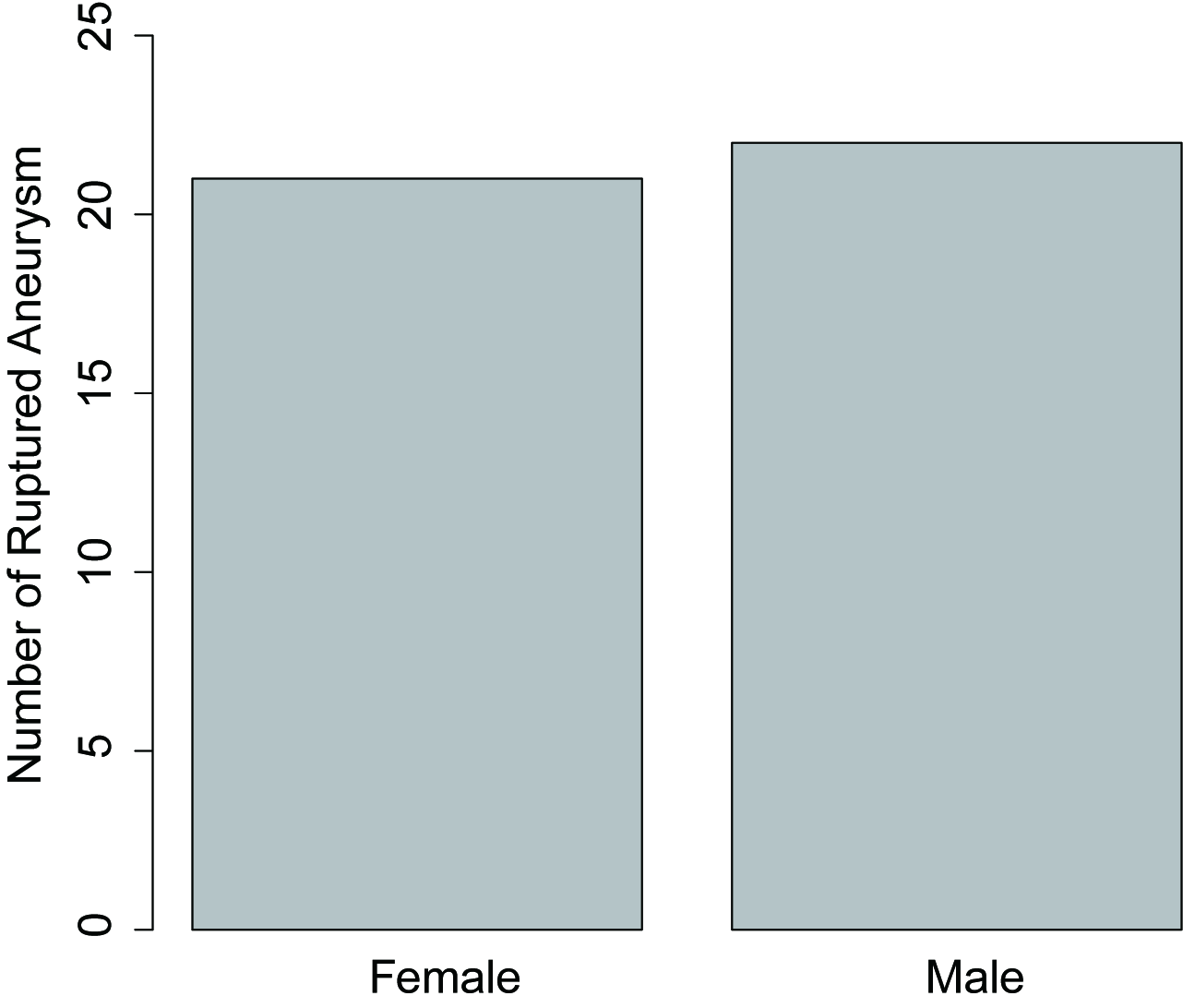

Supplement: Supplementary Figure 1 — The details of samples. [file Image_2.TIF]

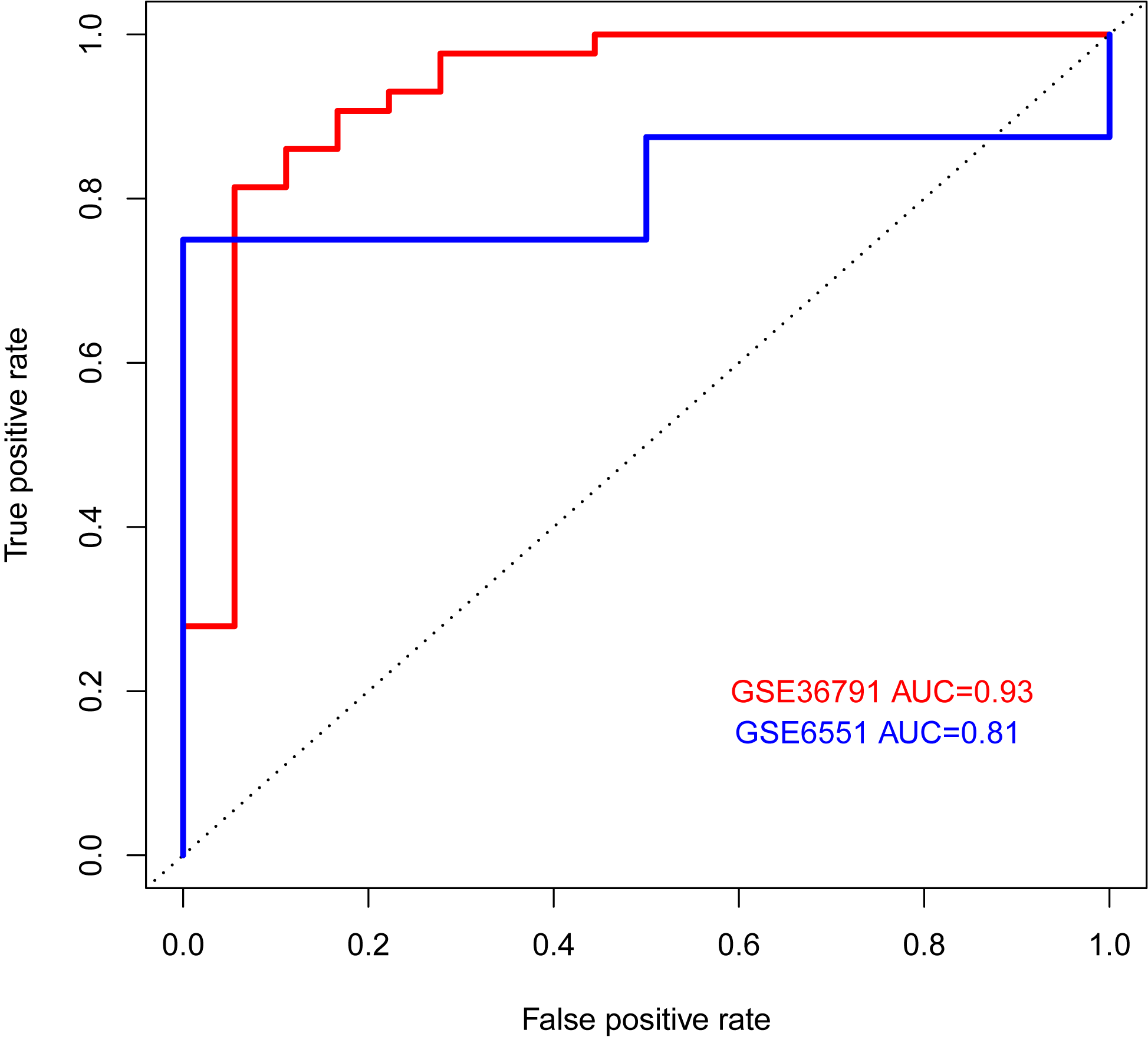

Supplement: Supplementary Figure 2 — The ROC curve. X-axis: false positive rate; Y-axis: true positive rate. The performance of the model was evaluated by the AUC value, and a larger AUC value within the range from 0 to 1 reflected a superior performance. [file Image_3.TIF]
