# Supplementary material for: A Two-Gene-Based Diagnostic Signature for Ruptured Intracranial Aneurysms
Source: Front Cardiovasc Med. 2021 Aug 13;8:671655. doi: 10.3389/fcvm.2021.671655 (PMC8414364; doi:10.3389/fcvm.2021.671655)
Supplement: Supplementary Table 1 — Clinical characteristics of the samples. [file Table_1.DOCX]

Table S1 Clinical characteristics of the samples.

|  | Ruptured aneurysms (n=43) | Controls (n=18) | P |
| --- | --- | --- | --- |
| Mean age (s.d.), years | 48.0 (13.9) | 46.1 (17.5) | 0.64 |
| Female, % | 48.8 | 72.2 | 0.16 |
| Hypertension, % | 44.2 | 22.2 | 0.15 |
| Smoking, % | 59.5 | 16.7 | 0.13 |
| Excessive drinking, % | 14.0 | 5.6 | 1.00 |
|  |  |  |  |
| Aneurysm's location | | | |
| Anterior circulation | 55 | — | — |
| Posterior circulation | 7 | — | — |
|  |  |  |  |
| Aneurysm's number (number of patients) | | | |
| 1 | 33 | — | — |
| 2 | 3 | — | — |
| 3 and more | 7 | — | — |
|  |  |  |  |
| Admission Hunt-Hess score (s.d.) | 2.8 (1.4) | — | — |
| Admission GCS score (s.d.) | 10.9 (4.9) | — | — |

GCS, Glasgow Coma Scale.
