# Supplementary material for: A Two-Gene-Based Diagnostic Signature for Ruptured Intracranial Aneurysms
Source: Front Cardiovasc Med. 2021 Aug 13;8:671655. doi: 10.3389/fcvm.2021.671655 (PMC8414364; doi:10.3389/fcvm.2021.671655)
Supplement: Supplementary Table 2 — Full list of significantly enriched GO terms of DEGs. [file Table_2.DOCX]

Table S2 Full list of significantly enriched GO terms of DEGs.

| ONTOLOGY | ID | Description | GeneRatio | BgRatio | pvalue | p.adjust | qvalue | geneID | Count |
| --- | --- | --- | --- | --- | --- | --- | --- | --- | --- |
| BP | GO:0042119 | neutrophil activation | 18/45 | 498/17653 | 8.02E-17 | 8.30E-14 | 6.40E-14 | 56729/728358/2992/820/3934/306/1991/8807/2352/6283/1669/57126/53831/7130/4318/1088/383/3240 | 18 |
| BP | GO:0043312 | neutrophil degranulation | 17/45 | 485/17653 | 1.19E-15 | 4.55E-13 | 3.51E-13 | 56729/728358/2992/820/3934/306/1991/2352/6283/1669/57126/53831/7130/4318/1088/383/3240 | 17 |
| BP | GO:0002283 | neutrophil activation involved in immune response | 17/45 | 488/17653 | 1.32E-15 | 4.55E-13 | 3.51E-13 | 56729/728358/2992/820/3934/306/1991/2352/6283/1669/57126/53831/7130/4318/1088/383/3240 | 17 |
| BP | GO:0002446 | neutrophil mediated immunity | 17/45 | 500/17653 | 1.97E-15 | 5.10E-13 | 3.93E-13 | 56729/728358/2992/820/3934/306/1991/2352/6283/1669/57126/53831/7130/4318/1088/383/3240 | 17 |
| BP | GO:0031640 | killing of cells of other organism | 7/45 | 57/17653 | 1.03E-10 | 1.78E-08 | 1.37E-08 | 728358/820/1991/1668/6283/1669/383 | 7 |
| BP | GO:0044364 | disruption of cells of other organism | 7/45 | 57/17653 | 1.03E-10 | 1.78E-08 | 1.37E-08 | 728358/820/1991/1668/6283/1669/383 | 7 |
| BP | GO:0050832 | defense response to fungus | 6/45 | 40/17653 | 6.98E-10 | 1.03E-07 | 7.96E-08 | 728358/1991/1668/6283/1669/383 | 6 |
| BP | GO:0001906 | cell killing | 8/45 | 141/17653 | 2.28E-09 | 2.95E-07 | 2.27E-07 | 728358/820/1991/1668/8807/6283/1669/383 | 8 |
| BP | GO:0009620 | response to fungus | 6/45 | 52/17653 | 3.62E-09 | 4.16E-07 | 3.21E-07 | 728358/1991/1668/6283/1669/383 | 6 |
| BP | GO:0061844 | antimicrobial humoral immune response mediated by antimicrobial peptide | 6/45 | 59/17653 | 7.90E-09 | 8.18E-07 | 6.31E-07 | 728358/820/1991/1668/6283/1669 | 6 |
| BP | GO:0019730 | antimicrobial humoral response | 7/45 | 108/17653 | 9.88E-09 | 9.29E-07 | 7.16E-07 | 728358/820/3934/1991/1668/6283/1669 | 7 |
| BP | GO:0051852 | disruption by host of symbiont cells | 4/45 | 13/17653 | 2.59E-08 | 2.23E-06 | 1.72E-06 | 728358/820/1991/383 | 4 |
| BP | GO:0042742 | defense response to bacterium | 9/45 | 293/17653 | 4.44E-08 | 3.53E-06 | 2.72E-06 | 728358/820/306/1991/1668/7100/6283/1669/3240 | 9 |
| BP | GO:0051818 | disruption of cells of other organism involved in symbiotic interaction | 4/45 | 17/17653 | 8.56E-08 | 6.33E-06 | 4.88E-06 | 728358/820/1991/383 | 4 |
| BP | GO:0019731 | antibacterial humoral response | 5/45 | 46/17653 | 1.09E-07 | 6.90E-06 | 5.32E-06 | 728358/820/1991/1668/1669 | 5 |
| BP | GO:0035821 | modification of morphology or physiology of other organism | 7/45 | 153/17653 | 1.10E-07 | 6.90E-06 | 5.32E-06 | 728358/820/1991/1668/6283/1669/383 | 7 |
| BP | GO:0032496 | response to lipopolysaccharide | 9/45 | 327/17653 | 1.13E-07 | 6.90E-06 | 5.32E-06 | 11213/728358/1991/1668/7100/1669/383/939/1236 | 9 |
| BP | GO:0002237 | response to molecule of bacterial origin | 9/45 | 345/17653 | 1.79E-07 | 1.03E-05 | 7.92E-06 | 11213/728358/1991/1668/7100/1669/383/939/1236 | 9 |
| BP | GO:0002227 | innate immune response in mucosa | 4/45 | 24/17653 | 3.77E-07 | 2.05E-05 | 1.58E-05 | 728358/820/1668/1669 | 4 |
| BP | GO:0006959 | humoral immune response | 8/45 | 329/17653 | 1.58E-06 | 7.96E-05 | 6.14E-05 | 728358/820/3934/1991/1668/6283/1669/1236 | 8 |
| BP | GO:0002385 | mucosal immune response | 4/45 | 34/17653 | 1.62E-06 | 7.96E-05 | 6.14E-05 | 728358/820/1668/1669 | 4 |
| BP | GO:0051673 | membrane disruption in other organism | 3/45 | 10/17653 | 1.83E-06 | 8.63E-05 | 6.65E-05 | 728358/1668/1669 | 3 |
| BP | GO:0002251 | organ or tissue specific immune response | 4/45 | 36/17653 | 2.04E-06 | 9.20E-05 | 7.09E-05 | 728358/820/1668/1669 | 4 |
| BP | GO:0051873 | killing by host of symbiont cells | 3/45 | 11/17653 | 2.52E-06 | 0.000109 | 8.37E-05 | 820/1991/383 | 3 |
| BP | GO:0051883 | killing of cells in other organism involved in symbiotic interaction | 3/45 | 15/17653 | 6.89E-06 | 0.000285 | 0.00022 | 820/1991/383 | 3 |
| BP | GO:0050900 | leukocyte migration | 8/45 | 470/17653 | 2.16E-05 | 0.000859 | 0.000662 | 728358/2994/1991/6283/57126/4318/1088/1236 | 8 |
| BP | GO:0051851 | modification by host of symbiont morphology or physiology | 4/45 | 71/17653 | 3.16E-05 | 0.001211 | 0.000934 | 728358/820/1991/383 | 4 |
| BP | GO:0051702 | interaction with symbiont | 4/45 | 75/17653 | 3.92E-05 | 0.00145 | 0.001118 | 728358/820/1991/383 | 4 |
| BP | GO:0050829 | defense response to Gram-negative bacterium | 4/45 | 84/17653 | 6.13E-05 | 0.002186 | 0.001685 | 728358/1991/1668/1669 | 4 |
| BP | GO:0071222 | cellular response to lipopolysaccharide | 5/45 | 174/17653 | 7.80E-05 | 0.002691 | 0.002074 | 728358/1668/7100/1669/383 | 5 |
| BP | GO:0050830 | defense response to Gram-positive bacterium | 4/45 | 92/17653 | 8.74E-05 | 0.002918 | 0.00225 | 728358/820/1668/1669 | 4 |
| BP | GO:0071219 | cellular response to molecule of bacterial origin | 5/45 | 182/17653 | 9.64E-05 | 0.003119 | 0.002404 | 728358/1668/7100/1669/383 | 5 |
| BP | GO:0051817 | modification of morphology or physiology of other organism involved in symbiotic interaction | 4/45 | 107/17653 | 0.000157 | 0.004924 | 0.003796 | 728358/820/1991/383 | 4 |
| BP | GO:0071216 | cellular response to biotic stimulus | 5/45 | 204/17653 | 0.000165 | 0.005012 | 0.003864 | 728358/1668/7100/1669/383 | 5 |
| BP | GO:0002526 | acute inflammatory response | 5/45 | 215/17653 | 0.00021 | 0.006217 | 0.004793 | 3557/1991/9332/3240/1236 | 5 |
| BP | GO:0006953 | acute-phase response | 3/45 | 48/17653 | 0.000247 | 0.007104 | 0.005476 | 3557/9332/3240 | 3 |
| BP | GO:0000302 | response to reactive oxygen species | 5/45 | 227/17653 | 0.00027 | 0.007562 | 0.00583 | 8807/4318/383/3240/1236 | 5 |
| BP | GO:0001960 | negative regulation of cytokine-mediated signaling pathway | 3/45 | 53/17653 | 0.000332 | 0.009034 | 0.006965 | 11213/3557/383 | 3 |
| BP | GO:0070673 | response to interleukin-18 | 2/45 | 11/17653 | 0.000344 | 0.00914 | 0.007046 | 8807/8809 | 2 |
| BP | GO:0060761 | negative regulation of response to cytokine stimulus | 3/45 | 57/17653 | 0.000411 | 0.010644 | 0.008205 | 11213/3557/383 | 3 |
| BP | GO:0044146 | negative regulation of growth of symbiont involved in interaction with host | 2/45 | 14/17653 | 0.000567 | 0.014315 | 0.011036 | 820/1991 | 2 |
| BP | GO:0044144 | modulation of growth of symbiont involved in interaction with host | 2/45 | 15/17653 | 0.000653 | 0.016098 | 0.01241 | 820/1991 | 2 |
| BP | GO:0035743 | CD4-positive, alpha-beta T cell cytokine production | 2/45 | 18/17653 | 0.000947 | 0.021787 | 0.016796 | 8807/383 | 2 |
| BP | GO:0044110 | growth involved in symbiotic interaction | 2/45 | 18/17653 | 0.000947 | 0.021787 | 0.016796 | 820/1991 | 2 |
| BP | GO:0044116 | growth of symbiont involved in interaction with host | 2/45 | 18/17653 | 0.000947 | 0.021787 | 0.016796 | 820/1991 | 2 |
| BP | GO:0015711 | organic anion transport | 6/45 | 460/17653 | 0.001043 | 0.023465 | 0.01809 | 759/2180/2352/7504/57198/383 | 6 |
| BP | GO:0031349 | positive regulation of defense response | 6/45 | 466/17653 | 0.001115 | 0.024552 | 0.018928 | 11213/8807/7100/6283/383/1236 | 6 |
| BP | GO:0042832 | defense response to protozoan | 2/45 | 20/17653 | 0.001173 | 0.025282 | 0.01949 | 728358/383 | 2 |
| BP | GO:0001562 | response to protozoan | 2/45 | 21/17653 | 0.001294 | 0.026264 | 0.020247 | 728358/383 | 2 |
| BP | GO:0002438 | acute inflammatory response to antigenic stimulus | 2/45 | 21/17653 | 0.001294 | 0.026264 | 0.020247 | 1991/1236 | 2 |
| BP | GO:0031348 | negative regulation of defense response | 4/45 | 187/17653 | 0.001294 | 0.026264 | 0.020247 | 11213/1991/7130/383 | 4 |
| BP | GO:0002367 | cytokine production involved in immune response | 3/45 | 85/17653 | 0.001321 | 0.026296 | 0.020272 | 11213/8807/383 | 3 |
| BP | GO:0002888 | positive regulation of myeloid leukocyte mediated immunity | 2/45 | 22/17653 | 0.001421 | 0.027748 | 0.021391 | 57126/383 | 2 |
| BP | GO:0001909 | leukocyte mediated cytotoxicity | 3/45 | 90/17653 | 0.001558 | 0.029469 | 0.022718 | 1991/8807/383 | 3 |
| BP | GO:0007159 | leukocyte cell-cell adhesion | 5/45 | 335/17653 | 0.001566 | 0.029469 | 0.022718 | 1991/57126/383/939/1236 | 5 |
| BP | GO:0002719 | negative regulation of cytokine production involved in immune response | 2/45 | 24/17653 | 0.001692 | 0.031276 | 0.024111 | 11213/383 | 2 |
| BP | GO:1990266 | neutrophil migration | 3/45 | 99/17653 | 0.002047 | 0.037162 | 0.028648 | 6283/57126/1236 | 3 |
| BP | GO:0002705 | positive regulation of leukocyte mediated immunity | 3/45 | 100/17653 | 0.002106 | 0.037584 | 0.028974 | 8807/57126/383 | 3 |
| BP | GO:0032609 | interferon-gamma production | 3/45 | 102/17653 | 0.002228 | 0.039093 | 0.030137 | 8807/8809/1236 | 3 |
| BP | GO:0043903 | regulation of symbiosis, encompassing mutualism through parasitism | 4/45 | 222/17653 | 0.002422 | 0.041769 | 0.0322 | 820/1991/6732/383 | 4 |
| BP | GO:0002440 | production of molecular mediator of immune response | 4/45 | 223/17653 | 0.002462 | 0.041769 | 0.0322 | 11213/1991/8807/383 | 4 |
| BP | GO:0030217 | T cell differentiation | 4/45 | 225/17653 | 0.002542 | 0.042443 | 0.03272 | 8809/939/64919/1236 | 4 |
| BP | GO:2000117 | negative regulation of cysteine-type endopeptidase activity | 3/45 | 111/17653 | 0.002833 | 0.046548 | 0.035884 | 8530/4318/939 | 3 |
| BP | GO:0022407 | regulation of cell-cell adhesion | 5/45 | 388/17653 | 0.002966 | 0.047965 | 0.036977 | 3557/1991/383/939/1236 | 5 |
| BP | GO:0048545 | response to steroid hormone | 5/45 | 391/17653 | 0.003066 | 0.048815 | 0.037632 | 728358/3557/306/1668/383 | 5 |
| BP | GO:0002701 | negative regulation of production of molecular mediator of immune response | 2/45 | 33/17653 | 0.00319 | 0.050033 | 0.038571 | 11213/383 | 2 |
| BP | GO:0002369 | T cell cytokine production | 2/45 | 34/17653 | 0.003384 | 0.052282 | 0.040305 | 8807/383 | 2 |
| BP | GO:0097530 | granulocyte migration | 3/45 | 119/17653 | 0.003448 | 0.052487 | 0.040463 | 6283/57126/1236 | 3 |
| BP | GO:0002755 | MyD88-dependent toll-like receptor signaling pathway | 2/45 | 36/17653 | 0.003788 | 0.056826 | 0.043808 | 11213/7100 | 2 |
| BP | GO:0001912 | positive regulation of leukocyte mediated cytotoxicity | 2/45 | 37/17653 | 0.003998 | 0.05912 | 0.045576 | 8807/383 | 2 |
| BP | GO:0002820 | negative regulation of adaptive immune response | 2/45 | 38/17653 | 0.004214 | 0.061426 | 0.047354 | 64092/383 | 2 |
| BP | GO:0042088 | T-helper 1 type immune response | 2/45 | 39/17653 | 0.004434 | 0.063744 | 0.049141 | 8807/8809 | 2 |
| BP | GO:0006979 | response to oxidative stress | 5/45 | 436/17653 | 0.004869 | 0.069036 | 0.053221 | 8807/4318/383/3240/1236 | 5 |
| BP | GO:0042542 | response to hydrogen peroxide | 3/45 | 138/17653 | 0.005219 | 0.072989 | 0.056268 | 8807/383/3240 | 3 |
| BP | GO:0002437 | inflammatory response to antigenic stimulus | 2/45 | 43/17653 | 0.005369 | 0.074057 | 0.057091 | 1991/1236 | 2 |
| BP | GO:0050777 | negative regulation of immune response | 3/45 | 141/17653 | 0.00554 | 0.074057 | 0.057091 | 11213/64092/383 | 3 |
| BP | GO:0051181 | cofactor transport | 2/45 | 44/17653 | 0.005616 | 0.074057 | 0.057091 | 3934/2352 | 2 |
| BP | GO:0051384 | response to glucocorticoid | 3/45 | 143/17653 | 0.00576 | 0.074057 | 0.057091 | 3557/306/383 | 3 |
| BP | GO:0002886 | regulation of myeloid leukocyte mediated immunity | 2/45 | 45/17653 | 0.005867 | 0.074057 | 0.057091 | 57126/383 | 2 |
| BP | GO:0032757 | positive regulation of interleukin-8 production | 2/45 | 45/17653 | 0.005867 | 0.074057 | 0.057091 | 1991/7100 | 2 |
| BP | GO:0045058 | T cell selection | 2/45 | 45/17653 | 0.005867 | 0.074057 | 0.057091 | 64919/1236 | 2 |
| BP | GO:0045824 | negative regulation of innate immune response | 2/45 | 45/17653 | 0.005867 | 0.074057 | 0.057091 | 11213/383 | 2 |
| BP | GO:0031343 | positive regulation of cell killing | 2/45 | 46/17653 | 0.006124 | 0.076367 | 0.058872 | 8807/383 | 2 |
| BP | GO:0042110 | T cell activation | 5/45 | 463/17653 | 0.006259 | 0.077114 | 0.059448 | 8809/383/939/64919/1236 | 5 |
| BP | GO:2000107 | negative regulation of leukocyte apoptotic process | 2/45 | 49/17653 | 0.006925 | 0.08432 | 0.065003 | 939/1236 | 2 |
| BP | GO:0032655 | regulation of interleukin-12 production | 2/45 | 50/17653 | 0.007202 | 0.085771 | 0.066122 | 11213/1236 | 2 |
| BP | GO:1903037 | regulation of leukocyte cell-cell adhesion | 4/45 | 302/17653 | 0.00721 | 0.085771 | 0.066122 | 1991/383/939/1236 | 4 |
| BP | GO:0002521 | leukocyte differentiation | 5/45 | 483/17653 | 0.007454 | 0.086403 | 0.066609 | 8809/4318/939/64919/1236 | 5 |
| BP | GO:0045123 | cellular extravasation | 2/45 | 51/17653 | 0.007484 | 0.086403 | 0.066609 | 1991/57126 | 2 |
| BP | GO:0030335 | positive regulation of cell migration | 5/45 | 486/17653 | 0.007646 | 0.086403 | 0.066609 | 306/1991/7130/4318/1236 | 5 |
| BP | GO:0032615 | interleukin-12 production | 2/45 | 52/17653 | 0.00777 | 0.086403 | 0.066609 | 11213/1236 | 2 |
| BP | GO:0015849 | organic acid transport | 4/45 | 309/17653 | 0.007804 | 0.086403 | 0.066609 | 2180/2352/7504/383 | 4 |
| BP | GO:0046942 | carboxylic acid transport | 4/45 | 309/17653 | 0.007804 | 0.086403 | 0.066609 | 2180/2352/7504/383 | 4 |
| BP | GO:0031960 | response to corticosteroid | 3/45 | 160/17653 | 0.007847 | 0.086403 | 0.066609 | 3557/306/383 | 3 |
| BP | GO:0034614 | cellular response to reactive oxygen species | 3/45 | 162/17653 | 0.008118 | 0.088448 | 0.068185 | 8807/4318/383 | 3 |
| BP | GO:0002460 | adaptive immune response based on somatic recombination of immune receptors built from immunoglobulin superfamily domains | 4/45 | 314/17653 | 0.008248 | 0.088926 | 0.068554 | 8807/8809/383/939 | 4 |
| BP | GO:0002703 | regulation of leukocyte mediated immunity | 3/45 | 168/17653 | 0.008965 | 0.095657 | 0.073743 | 8807/57126/383 | 3 |
| BP | GO:0046677 | response to antibiotic | 4/45 | 326/17653 | 0.009381 | 0.097724 | 0.075337 | 8807/383/3240/939 | 4 |
| BP | GO:0001959 | regulation of cytokine-mediated signaling pathway | 3/45 | 172/17653 | 0.009557 | 0.097724 | 0.075337 | 11213/3557/383 | 3 |
| BP | GO:0043901 | negative regulation of multi-organism process | 3/45 | 172/17653 | 0.009557 | 0.097724 | 0.075337 | 820/1991/6732 | 3 |
| BP | GO:0030520 | intracellular estrogen receptor signaling pathway | 2/45 | 58/17653 | 0.009593 | 0.097724 | 0.075337 | 728358/1668 | 2 |
| BP | GO:2000377 | regulation of reactive oxygen species metabolic process | 3/45 | 173/17653 | 0.009709 | 0.097724 | 0.075337 | 7100/57126/3240 | 3 |
| BP | GO:0030098 | lymphocyte differentiation | 4/45 | 330/17653 | 0.00978 | 0.097724 | 0.075337 | 8809/939/64919/1236 | 4 |
| BP | GO:0071466 | cellular response to xenobiotic stimulus | 3/45 | 174/17653 | 0.009862 | 0.097724 | 0.075337 | 2180/6283/383 | 3 |
| BP | GO:0001910 | regulation of leukocyte mediated cytotoxicity | 2/45 | 59/17653 | 0.009914 | 0.097724 | 0.075337 | 8807/383 | 2 |
| BP | GO:0002699 | positive regulation of immune effector process | 3/45 | 175/17653 | 0.010016 | 0.097797 | 0.075393 | 8807/57126/383 | 3 |
| BP | GO:0034121 | regulation of toll-like receptor signaling pathway | 2/45 | 60/17653 | 0.010239 | 0.098984 | 0.076308 | 11213/7100 | 2 |
| BP | GO:0097529 | myeloid leukocyte migration | 3/45 | 177/17653 | 0.010329 | 0.098984 | 0.076308 | 6283/57126/1236 | 3 |
| BP | GO:0060759 | regulation of response to cytokine stimulus | 3/45 | 180/17653 | 0.010809 | 0.102634 | 0.079122 | 11213/3557/383 | 3 |
| BP | GO:0032677 | regulation of interleukin-8 production | 2/45 | 63/17653 | 0.011244 | 0.105792 | 0.081556 | 1991/7100 | 2 |
| BP | GO:0046686 | response to cadmium ion | 2/45 | 64/17653 | 0.011588 | 0.108047 | 0.083295 | 4318/383 | 2 |
| BP | GO:0033077 | T cell differentiation in thymus | 2/45 | 65/17653 | 0.011936 | 0.110304 | 0.085035 | 64919/1236 | 2 |
| BP | GO:0002718 | regulation of cytokine production involved in immune response | 2/45 | 66/17653 | 0.01229 | 0.112563 | 0.086776 | 11213/383 | 2 |
| BP | GO:0015893 | drug transport | 3/45 | 195/17653 | 0.013402 | 0.121674 | 0.0938 | 56729/2352/383 | 3 |
| BP | GO:0050663 | cytokine secretion | 3/45 | 196/17653 | 0.013586 | 0.122275 | 0.094264 | 7100/6283/1236 | 3 |
| BP | GO:0032637 | interleukin-8 production | 2/45 | 70/17653 | 0.013748 | 0.122663 | 0.094562 | 1991/7100 | 2 |
| BP | GO:0031341 | regulation of cell killing | 2/45 | 71/17653 | 0.014123 | 0.124939 | 0.096317 | 8807/383 | 2 |
| BP | GO:0030595 | leukocyte chemotaxis | 3/45 | 202/17653 | 0.014723 | 0.129139 | 0.099555 | 728358/6283/1236 | 3 |
| BP | GO:0009988 | cell-cell recognition | 2/45 | 73/17653 | 0.014888 | 0.129491 | 0.099826 | 2352/1236 | 2 |
| BP | GO:0043900 | regulation of multi-organism process | 4/45 | 391/17653 | 0.017283 | 0.149068 | 0.114918 | 820/1991/6732/383 | 4 |
| BP | GO:0002456 | T cell mediated immunity | 2/45 | 81/17653 | 0.018121 | 0.153733 | 0.118515 | 8807/383 | 2 |
| BP | GO:0070542 | response to fatty acid | 2/45 | 81/17653 | 0.018121 | 0.153733 | 0.118515 | 2180/1236 | 2 |
| BP | GO:1903039 | positive regulation of leukocyte cell-cell adhesion | 3/45 | 224/17653 | 0.019344 | 0.15864 | 0.122298 | 1991/939/1236 | 3 |
| BP | GO:0002697 | regulation of immune effector process | 4/45 | 405/17653 | 0.019402 | 0.15864 | 0.122298 | 11213/8807/57126/383 | 4 |
| BP | GO:2000106 | regulation of leukocyte apoptotic process | 2/45 | 84/17653 | 0.019403 | 0.15864 | 0.122298 | 939/1236 | 2 |
| BP | GO:0048661 | positive regulation of smooth muscle cell proliferation | 2/45 | 86/17653 | 0.020279 | 0.15864 | 0.122298 | 1991/4318 | 2 |
| BP | GO:0001819 | positive regulation of cytokine production | 4/45 | 411/17653 | 0.020357 | 0.15864 | 0.122298 | 1991/7100/8809/1236 | 4 |
| BP | GO:0050727 | regulation of inflammatory response | 4/45 | 411/17653 | 0.020357 | 0.15864 | 0.122298 | 1991/6283/7130/1236 | 4 |
| BP | GO:0097237 | cellular response to toxic substance | 3/45 | 231/17653 | 0.020965 | 0.15864 | 0.122298 | 8807/383/3240 | 3 |
| BP | GO:0030593 | neutrophil chemotaxis | 2/45 | 88/17653 | 0.021171 | 0.15864 | 0.122298 | 6283/1236 | 2 |
| BP | GO:0060333 | interferon-gamma-mediated signaling pathway | 2/45 | 89/17653 | 0.021623 | 0.15864 | 0.122298 | 2209/383 | 2 |
| BP | GO:0009755 | hormone-mediated signaling pathway | 3/45 | 234/17653 | 0.021682 | 0.15864 | 0.122298 | 728358/2180/1668 | 3 |
| BP | GO:0032649 | regulation of interferon-gamma production | 2/45 | 91/17653 | 0.022539 | 0.15864 | 0.122298 | 8809/1236 | 2 |
| BP | GO:0043154 | negative regulation of cysteine-type endopeptidase activity involved in apoptotic process | 2/45 | 91/17653 | 0.022539 | 0.15864 | 0.122298 | 4318/939 | 2 |
| BP | GO:0046632 | alpha-beta T cell differentiation | 2/45 | 91/17653 | 0.022539 | 0.15864 | 0.122298 | 8809/64919 | 2 |
| BP | GO:2001023 | regulation of response to drug | 2/45 | 91/17653 | 0.022539 | 0.15864 | 0.122298 | 56729/383 | 2 |
| BP | GO:2000379 | positive regulation of reactive oxygen species metabolic process | 2/45 | 92/17653 | 0.023003 | 0.15864 | 0.122298 | 7100/57126 | 2 |
| BP | GO:0045088 | regulation of innate immune response | 4/45 | 427/17653 | 0.023045 | 0.15864 | 0.122298 | 11213/8807/7100/383 | 4 |
| BP | GO:0070301 | cellular response to hydrogen peroxide | 2/45 | 93/17653 | 0.023471 | 0.15864 | 0.122298 | 8807/383 | 2 |
| BP | GO:0022617 | extracellular matrix disassembly | 2/45 | 94/17653 | 0.023943 | 0.15864 | 0.122298 | 1991/4318 | 2 |
| BP | GO:0072676 | lymphocyte migration | 2/45 | 94/17653 | 0.023943 | 0.15864 | 0.122298 | 728358/1236 | 2 |
| BP | GO:0001818 | negative regulation of cytokine production | 3/45 | 245/17653 | 0.024425 | 0.15864 | 0.122298 | 11213/1991/383 | 3 |
| BP | GO:0051091 | positive regulation of DNA binding transcription factor activity | 3/45 | 245/17653 | 0.024425 | 0.15864 | 0.122298 | 11213/306/6283 | 3 |
| BP | GO:0001553 | luteinization | 1/45 | 10/17653 | 0.025207 | 0.15864 | 0.122298 | 56729 | 1 |
| BP | GO:0001768 | establishment of T cell polarity | 1/45 | 10/17653 | 0.025207 | 0.15864 | 0.122298 | 1236 | 1 |
| BP | GO:0002863 | positive regulation of inflammatory response to antigenic stimulus | 1/45 | 10/17653 | 0.025207 | 0.15864 | 0.122298 | 1236 | 1 |
| BP | GO:0010960 | magnesium ion homeostasis | 1/45 | 10/17653 | 0.025207 | 0.15864 | 0.122298 | 7504 | 1 |
| BP | GO:0021877 | forebrain neuron fate commitment | 1/45 | 10/17653 | 0.025207 | 0.15864 | 0.122298 | 64919 | 1 |
| BP | GO:0034115 | negative regulation of heterotypic cell-cell adhesion | 1/45 | 10/17653 | 0.025207 | 0.15864 | 0.122298 | 3557 | 1 |
| BP | GO:0045060 | negative thymic T cell selection | 1/45 | 10/17653 | 0.025207 | 0.15864 | 0.122298 | 1236 | 1 |
| BP | GO:0045217 | cell-cell junction maintenance | 1/45 | 10/17653 | 0.025207 | 0.15864 | 0.122298 | 57126 | 1 |
| BP | GO:0051547 | regulation of keratinocyte migration | 1/45 | 10/17653 | 0.025207 | 0.15864 | 0.122298 | 4318 | 1 |
| BP | GO:0070587 | regulation of cell-cell adhesion involved in gastrulation | 1/45 | 10/17653 | 0.025207 | 0.15864 | 0.122298 | 3557 | 1 |
| BP | GO:0072672 | neutrophil extravasation | 1/45 | 10/17653 | 0.025207 | 0.15864 | 0.122298 | 57126 | 1 |
| BP | GO:0097048 | dendritic cell apoptotic process | 1/45 | 10/17653 | 0.025207 | 0.15864 | 0.122298 | 1236 | 1 |
| BP | GO:1904996 | positive regulation of leukocyte adhesion to vascular endothelial cell | 1/45 | 10/17653 | 0.025207 | 0.15864 | 0.122298 | 1991 | 1 |
| BP | GO:2000551 | regulation of T-helper 2 cell cytokine production | 1/45 | 10/17653 | 0.025207 | 0.15864 | 0.122298 | 383 | 1 |
| BP | GO:2000668 | regulation of dendritic cell apoptotic process | 1/45 | 10/17653 | 0.025207 | 0.15864 | 0.122298 | 1236 | 1 |
| BP | GO:2000116 | regulation of cysteine-type endopeptidase activity | 3/45 | 249/17653 | 0.025467 | 0.15864 | 0.122298 | 8530/4318/939 | 3 |
| BP | GO:0010951 | negative regulation of endopeptidase activity | 3/45 | 251/17653 | 0.025997 | 0.15864 | 0.122298 | 8530/4318/939 | 3 |
| BP | GO:0070498 | interleukin-1-mediated signaling pathway | 2/45 | 100/17653 | 0.026855 | 0.15864 | 0.122298 | 11213/3557 | 2 |
| BP | GO:0072593 | reactive oxygen species metabolic process | 3/45 | 256/17653 | 0.027348 | 0.15864 | 0.122298 | 7100/57126/3240 | 3 |
| BP | GO:0022409 | positive regulation of cell-cell adhesion | 3/45 | 257/17653 | 0.027622 | 0.15864 | 0.122298 | 1991/939/1236 | 3 |
| BP | GO:0001767 | establishment of lymphocyte polarity | 1/45 | 11/17653 | 0.027694 | 0.15864 | 0.122298 | 1236 | 1 |
| BP | GO:0002523 | leukocyte migration involved in inflammatory response | 1/45 | 11/17653 | 0.027694 | 0.15864 | 0.122298 | 1991 | 1 |
| BP | GO:0032494 | response to peptidoglycan | 1/45 | 11/17653 | 0.027694 | 0.15864 | 0.122298 | 11213 | 1 |
| BP | GO:0042178 | xenobiotic catabolic process | 1/45 | 11/17653 | 0.027694 | 0.15864 | 0.122298 | 2180 | 1 |
| BP | GO:0043383 | negative T cell selection | 1/45 | 11/17653 | 0.027694 | 0.15864 | 0.122298 | 1236 | 1 |
| BP | GO:0045073 | regulation of chemokine biosynthetic process | 1/45 | 11/17653 | 0.027694 | 0.15864 | 0.122298 | 1991 | 1 |
| BP | GO:0046007 | negative regulation of activated T cell proliferation | 1/45 | 11/17653 | 0.027694 | 0.15864 | 0.122298 | 383 | 1 |
| BP | GO:0070586 | cell-cell adhesion involved in gastrulation | 1/45 | 11/17653 | 0.027694 | 0.15864 | 0.122298 | 3557 | 1 |
| BP | GO:1902563 | regulation of neutrophil activation | 1/45 | 11/17653 | 0.027694 | 0.15864 | 0.122298 | 57126 | 1 |
| BP | GO:1905941 | positive regulation of gonad development | 1/45 | 11/17653 | 0.027694 | 0.15864 | 0.122298 | 56729 | 1 |
| BP | GO:0071887 | leukocyte apoptotic process | 2/45 | 102/17653 | 0.027857 | 0.15864 | 0.122298 | 939/1236 | 2 |
| BP | GO:0071383 | cellular response to steroid hormone stimulus | 3/45 | 260/17653 | 0.028455 | 0.15864 | 0.122298 | 728358/1668/383 | 3 |
| BP | GO:0051249 | regulation of lymphocyte activation | 4/45 | 457/17653 | 0.028651 | 0.15864 | 0.122298 | 64092/383/939/1236 | 4 |
| BP | GO:0002698 | negative regulation of immune effector process | 2/45 | 105/17653 | 0.029386 | 0.15864 | 0.122298 | 11213/383 | 2 |
| BP | GO:0071621 | granulocyte chemotaxis | 2/45 | 105/17653 | 0.029386 | 0.15864 | 0.122298 | 6283/1236 | 2 |
| BP | GO:0010466 | negative regulation of peptidase activity | 3/45 | 264/17653 | 0.029586 | 0.15864 | 0.122298 | 8530/4318/939 | 3 |
| BP | GO:0000050 | urea cycle | 1/45 | 12/17653 | 0.030174 | 0.15864 | 0.122298 | 383 | 1 |
| BP | GO:0001660 | fever generation | 1/45 | 12/17653 | 0.030174 | 0.15864 | 0.122298 | 3557 | 1 |
| BP | GO:0002864 | regulation of acute inflammatory response to antigenic stimulus | 1/45 | 12/17653 | 0.030174 | 0.15864 | 0.122298 | 1236 | 1 |
| BP | GO:0003334 | keratinocyte development | 1/45 | 12/17653 | 0.030174 | 0.15864 | 0.122298 | 64919 | 1 |
| BP | GO:0006527 | arginine catabolic process | 1/45 | 12/17653 | 0.030174 | 0.15864 | 0.122298 | 383 | 1 |
| BP | GO:0009635 | response to herbicide | 1/45 | 12/17653 | 0.030174 | 0.15864 | 0.122298 | 383 | 1 |
| BP | GO:0010935 | regulation of macrophage cytokine production | 1/45 | 12/17653 | 0.030174 | 0.15864 | 0.122298 | 11213 | 1 |
| BP | GO:0033197 | response to vitamin E | 1/45 | 12/17653 | 0.030174 | 0.15864 | 0.122298 | 383 | 1 |
| BP | GO:0034331 | cell junction maintenance | 1/45 | 12/17653 | 0.030174 | 0.15864 | 0.122298 | 57126 | 1 |
| BP | GO:0035092 | sperm chromatin condensation | 1/45 | 12/17653 | 0.030174 | 0.15864 | 0.122298 | 6732 | 1 |
| BP | GO:0038110 | interleukin-2-mediated signaling pathway | 1/45 | 12/17653 | 0.030174 | 0.15864 | 0.122298 | 3560 | 1 |
| BP | GO:0042033 | chemokine biosynthetic process | 1/45 | 12/17653 | 0.030174 | 0.15864 | 0.122298 | 1991 | 1 |
| BP | GO:0045414 | regulation of interleukin-8 biosynthetic process | 1/45 | 12/17653 | 0.030174 | 0.15864 | 0.122298 | 1991 | 1 |
| BP | GO:0050755 | chemokine metabolic process | 1/45 | 12/17653 | 0.030174 | 0.15864 | 0.122298 | 1991 | 1 |
| BP | GO:0051712 | positive regulation of killing of cells of other organism | 1/45 | 12/17653 | 0.030174 | 0.15864 | 0.122298 | 383 | 1 |
| BP | GO:1901678 | iron coordination entity transport | 1/45 | 12/17653 | 0.030174 | 0.15864 | 0.122298 | 3934 | 1 |
| BP | GO:0060326 | cell chemotaxis | 3/45 | 273/17653 | 0.032218 | 0.15864 | 0.122298 | 728358/6283/1236 | 3 |
| BP | GO:0001771 | immunological synapse formation | 1/45 | 13/17653 | 0.032648 | 0.15864 | 0.122298 | 1236 | 1 |
| BP | GO:0002693 | positive regulation of cellular extravasation | 1/45 | 13/17653 | 0.032648 | 0.15864 | 0.122298 | 1991 | 1 |
| BP | GO:0015802 | basic amino acid transport | 1/45 | 13/17653 | 0.032648 | 0.15864 | 0.122298 | 383 | 1 |
| BP | GO:0031274 | positive regulation of pseudopodium assembly | 1/45 | 13/17653 | 0.032648 | 0.15864 | 0.122298 | 1236 | 1 |
| BP | GO:0032536 | regulation of cell projection size | 1/45 | 13/17653 | 0.032648 | 0.15864 | 0.122298 | 7504 | 1 |
| BP | GO:0035723 | interleukin-15-mediated signaling pathway | 1/45 | 13/17653 | 0.032648 | 0.15864 | 0.122298 | 3560 | 1 |
| BP | GO:0035745 | T-helper 2 cell cytokine production | 1/45 | 13/17653 | 0.032648 | 0.15864 | 0.122298 | 383 | 1 |
| BP | GO:0036109 | alpha-linolenic acid metabolic process | 1/45 | 13/17653 | 0.032648 | 0.15864 | 0.122298 | 2180 | 1 |
| BP | GO:0042228 | interleukin-8 biosynthetic process | 1/45 | 13/17653 | 0.032648 | 0.15864 | 0.122298 | 1991 | 1 |
| BP | GO:0044130 | negative regulation of growth of symbiont in host | 1/45 | 13/17653 | 0.032648 | 0.15864 | 0.122298 | 1991 | 1 |
| BP | GO:0045579 | positive regulation of B cell differentiation | 1/45 | 13/17653 | 0.032648 | 0.15864 | 0.122298 | 939 | 1 |
| BP | GO:0050862 | positive regulation of T cell receptor signaling pathway | 1/45 | 13/17653 | 0.032648 | 0.15864 | 0.122298 | 1236 | 1 |
| BP | GO:0051709 | regulation of killing of cells of other organism | 1/45 | 13/17653 | 0.032648 | 0.15864 | 0.122298 | 383 | 1 |
| BP | GO:0071350 | cellular response to interleukin-15 | 1/45 | 13/17653 | 0.032648 | 0.15864 | 0.122298 | 3560 | 1 |
| BP | GO:0071352 | cellular response to interleukin-2 | 1/45 | 13/17653 | 0.032648 | 0.15864 | 0.122298 | 3560 | 1 |
| BP | GO:2000194 | regulation of female gonad development | 1/45 | 13/17653 | 0.032648 | 0.15864 | 0.122298 | 56729 | 1 |
| BP | GO:2001044 | regulation of integrin-mediated signaling pathway | 1/45 | 13/17653 | 0.032648 | 0.15864 | 0.122298 | 57126 | 1 |
| BP | GO:0009410 | response to xenobiotic stimulus | 3/45 | 277/17653 | 0.033427 | 0.160816 | 0.123975 | 2180/6283/383 | 3 |
| BP | GO:0002700 | regulation of production of molecular mediator of immune response | 2/45 | 115/17653 | 0.034721 | 0.160816 | 0.123975 | 11213/383 | 2 |
| BP | GO:0002829 | negative regulation of type 2 immune response | 1/45 | 14/17653 | 0.035115 | 0.160816 | 0.123975 | 383 | 1 |
| BP | GO:0010310 | regulation of hydrogen peroxide metabolic process | 1/45 | 14/17653 | 0.035115 | 0.160816 | 0.123975 | 3240 | 1 |
| BP | GO:0010934 | macrophage cytokine production | 1/45 | 14/17653 | 0.035115 | 0.160816 | 0.123975 | 11213 | 1 |
| BP | GO:0019627 | urea metabolic process | 1/45 | 14/17653 | 0.035115 | 0.160816 | 0.123975 | 383 | 1 |
| BP | GO:0020027 | hemoglobin metabolic process | 1/45 | 14/17653 | 0.035115 | 0.160816 | 0.123975 | 51327 | 1 |
| BP | GO:0031272 | regulation of pseudopodium assembly | 1/45 | 14/17653 | 0.035115 | 0.160816 | 0.123975 | 1236 | 1 |
| BP | GO:0032930 | positive regulation of superoxide anion generation | 1/45 | 14/17653 | 0.035115 | 0.160816 | 0.123975 | 57126 | 1 |
| BP | GO:0033151 | V(D)J recombination | 1/45 | 14/17653 | 0.035115 | 0.160816 | 0.123975 | 64919 | 1 |
| BP | GO:0044126 | regulation of growth of symbiont in host | 1/45 | 14/17653 | 0.035115 | 0.160816 | 0.123975 | 1991 | 1 |
| BP | GO:0070669 | response to interleukin-2 | 1/45 | 14/17653 | 0.035115 | 0.160816 | 0.123975 | 3560 | 1 |
| BP | GO:0070672 | response to interleukin-15 | 1/45 | 14/17653 | 0.035115 | 0.160816 | 0.123975 | 3560 | 1 |
| BP | GO:0046631 | alpha-beta T cell activation | 2/45 | 116/17653 | 0.035274 | 0.160831 | 0.123987 | 8809/64919 | 2 |
| BP | GO:0034599 | cellular response to oxidative stress | 3/45 | 286/17653 | 0.036231 | 0.16447 | 0.126792 | 8807/4318/383 | 3 |
| BP | GO:0032103 | positive regulation of response to external stimulus | 3/45 | 289/17653 | 0.037192 | 0.164797 | 0.127044 | 6283/383/1236 | 3 |
| BP | GO:0006805 | xenobiotic metabolic process | 2/45 | 120/17653 | 0.03752 | 0.164797 | 0.127044 | 2180/6283 | 2 |
| BP | GO:0032635 | interleukin-6 production | 2/45 | 120/17653 | 0.03752 | 0.164797 | 0.127044 | 11213/8807 | 2 |
| BP | GO:0002922 | positive regulation of humoral immune response | 1/45 | 15/17653 | 0.037577 | 0.164797 | 0.127044 | 1236 | 1 |
| BP | GO:0048521 | negative regulation of behavior | 1/45 | 15/17653 | 0.037577 | 0.164797 | 0.127044 | 56729 | 1 |
| BP | GO:0048569 | post-embryonic animal organ development | 1/45 | 15/17653 | 0.037577 | 0.164797 | 0.127044 | 64919 | 1 |
| BP | GO:0051546 | keratinocyte migration | 1/45 | 15/17653 | 0.037577 | 0.164797 | 0.127044 | 4318 | 1 |
| BP | GO:0071941 | nitrogen cycle metabolic process | 1/45 | 15/17653 | 0.037577 | 0.164797 | 0.127044 | 383 | 1 |
| BP | GO:0002687 | positive regulation of leukocyte migration | 2/45 | 123/17653 | 0.039239 | 0.169809 | 0.130908 | 1991/1236 | 2 |
| BP | GO:0002706 | regulation of lymphocyte mediated immunity | 2/45 | 124/17653 | 0.039819 | 0.169809 | 0.130908 | 8807/383 | 2 |
| BP | GO:0032963 | collagen metabolic process | 2/45 | 124/17653 | 0.039819 | 0.169809 | 0.130908 | 4318/383 | 2 |
| BP | GO:0046330 | positive regulation of JNK cascade | 2/45 | 124/17653 | 0.039819 | 0.169809 | 0.130908 | 939/1236 | 2 |
| BP | GO:0050729 | positive regulation of inflammatory response | 2/45 | 124/17653 | 0.039819 | 0.169809 | 0.130908 | 6283/1236 | 2 |
| BP | GO:0002643 | regulation of tolerance induction | 1/45 | 16/17653 | 0.040032 | 0.169809 | 0.130908 | 11213 | 1 |
| BP | GO:0031269 | pseudopodium assembly | 1/45 | 16/17653 | 0.040032 | 0.169809 | 0.130908 | 1236 | 1 |
| BP | GO:0032695 | negative regulation of interleukin-12 production | 1/45 | 16/17653 | 0.040032 | 0.169809 | 0.130908 | 11213 | 1 |
| BP | GO:0002577 | regulation of antigen processing and presentation | 1/45 | 17/17653 | 0.042482 | 0.172426 | 0.132925 | 1236 | 1 |
| BP | GO:0002710 | negative regulation of T cell mediated immunity | 1/45 | 17/17653 | 0.042482 | 0.172426 | 0.132925 | 383 | 1 |
| BP | GO:0007342 | fusion of sperm to egg plasma membrane involved in single fertilization | 1/45 | 17/17653 | 0.042482 | 0.172426 | 0.132925 | 2352 | 1 |
| BP | GO:0010042 | response to manganese ion | 1/45 | 17/17653 | 0.042482 | 0.172426 | 0.132925 | 383 | 1 |
| BP | GO:0021756 | striatum development | 1/45 | 17/17653 | 0.042482 | 0.172426 | 0.132925 | 64919 | 1 |
| BP | GO:0031268 | pseudopodium organization | 1/45 | 17/17653 | 0.042482 | 0.172426 | 0.132925 | 1236 | 1 |
| BP | GO:0032717 | negative regulation of interleukin-8 production | 1/45 | 17/17653 | 0.042482 | 0.172426 | 0.132925 | 1991 | 1 |
| BP | GO:0033189 | response to vitamin A | 1/45 | 17/17653 | 0.042482 | 0.172426 | 0.132925 | 383 | 1 |
| BP | GO:0043651 | linoleic acid metabolic process | 1/45 | 17/17653 | 0.042482 | 0.172426 | 0.132925 | 2180 | 1 |
| BP | GO:0044117 | growth of symbiont in host | 1/45 | 17/17653 | 0.042482 | 0.172426 | 0.132925 | 1991 | 1 |
| BP | GO:0045063 | T-helper 1 cell differentiation | 1/45 | 17/17653 | 0.042482 | 0.172426 | 0.132925 | 8809 | 1 |
| BP | GO:0050728 | negative regulation of inflammatory response | 2/45 | 130/17653 | 0.043366 | 0.175327 | 0.135162 | 1991/7130 | 2 |
| BP | GO:0002819 | regulation of adaptive immune response | 2/45 | 131/17653 | 0.043968 | 0.175461 | 0.135265 | 64092/383 | 2 |
| BP | GO:0009615 | response to virus | 3/45 | 310/17653 | 0.044286 | 0.175461 | 0.135265 | 11213/728358/1668 | 3 |
| BP | GO:0051250 | negative regulation of lymphocyte activation | 2/45 | 132/17653 | 0.044574 | 0.175461 | 0.135265 | 64092/383 | 2 |
| BP | GO:0032682 | negative regulation of chemokine production | 1/45 | 18/17653 | 0.044925 | 0.175461 | 0.135265 | 1991 | 1 |
| BP | GO:0032928 | regulation of superoxide anion generation | 1/45 | 18/17653 | 0.044925 | 0.175461 | 0.135265 | 57126 | 1 |
| BP | GO:0035338 | long-chain fatty-acyl-CoA biosynthetic process | 1/45 | 18/17653 | 0.044925 | 0.175461 | 0.135265 | 2180 | 1 |
| BP | GO:0051195 | negative regulation of cofactor metabolic process | 1/45 | 18/17653 | 0.044925 | 0.175461 | 0.135265 | 3240 | 1 |
| BP | GO:0072337 | modified amino acid transport | 1/45 | 18/17653 | 0.044925 | 0.175461 | 0.135265 | 2352 | 1 |
| BP | GO:2000831 | regulation of steroid hormone secretion | 1/45 | 18/17653 | 0.044925 | 0.175461 | 0.135265 | 56729 | 1 |
| BP | GO:0002449 | lymphocyte mediated immunity | 3/45 | 315/17653 | 0.046069 | 0.179252 | 0.138188 | 8807/383/939 | 3 |
| BP | GO:0002224 | toll-like receptor signaling pathway | 2/45 | 135/17653 | 0.046409 | 0.1799 | 0.138687 | 11213/7100 | 2 |
| BP | GO:0010560 | positive regulation of glycoprotein biosynthetic process | 1/45 | 19/17653 | 0.047362 | 0.180219 | 0.138933 | 1236 | 1 |
| BP | GO:0045061 | thymic T cell selection | 1/45 | 19/17653 | 0.047362 | 0.180219 | 0.138933 | 1236 | 1 |
| BP | GO:0070233 | negative regulation of T cell apoptotic process | 1/45 | 19/17653 | 0.047362 | 0.180219 | 0.138933 | 939 | 1 |
| BP | GO:0071731 | response to nitric oxide | 1/45 | 19/17653 | 0.047362 | 0.180219 | 0.138933 | 1236 | 1 |
| BP | GO:1905939 | regulation of gonad development | 1/45 | 19/17653 | 0.047362 | 0.180219 | 0.138933 | 56729 | 1 |
| BP | GO:0006865 | amino acid transport | 2/45 | 137/17653 | 0.047648 | 0.180643 | 0.13926 | 7504/383 | 2 |
| BP | GO:0050863 | regulation of T cell activation | 3/45 | 321/17653 | 0.048254 | 0.181677 | 0.140057 | 383/939/1236 | 3 |
| BP | GO:0030518 | intracellular steroid hormone receptor signaling pathway | 2/45 | 138/17653 | 0.048272 | 0.181677 | 0.140057 | 728358/1668 | 2 |
| BP | GO:0031649 | heat generation | 1/45 | 20/17653 | 0.049793 | 0.185724 | 0.143177 | 3557 | 1 |
| BP | GO:0034123 | positive regulation of toll-like receptor signaling pathway | 1/45 | 20/17653 | 0.049793 | 0.185724 | 0.143177 | 7100 | 1 |
