# Supplementary material for: A Two-Gene-Based Diagnostic Signature for Ruptured Intracranial Aneurysms
Source: Front Cardiovasc Med. 2021 Aug 13;8:671655. doi: 10.3389/fcvm.2021.671655 (PMC8414364; doi:10.3389/fcvm.2021.671655)
Supplement: Supplementary Table 3 — Full list of KEGG pathways of DEGs. [file Table_3.DOCX]

| ID | Description | BgRatio | pvalue | p.adjust | qvalue | geneID | Count |
| --- | --- | --- | --- | --- | --- | --- | --- |
| hsa05150 | Staphylococcus aureus infection | 96/8016 | 1.45E-05 | 0.000880262 | 0.000807032 | 728358/820/2209/1668/1669 | 5 |
| hsa05202 | Transcriptional misregulation in cancer | 186/8016 | 2.84E-05 | 0.000880262 | 0.000807032 | 2209/1991/1668/4318/64919/3560 | 6 |
| hsa04061 | Viral protein interaction with cytokine and cytokine receptor | 100/8016 | 0.000321011 | 0.005529471 | 0.005069464 | 8807/8809/1236/3560 | 4 |
| hsa04060 | Cytokine-cytokine receptor interaction | 294/8016 | 0.00035674 | 0.005529471 | 0.005069464 | 3557/8807/8809/939/1236/3560 | 6 |
| hsa05321 | Inflammatory bowel disease (IBD) | 65/8016 | 0.001295368 | 0.016062565 | 0.014726291 | 8807/7100/8809 | 3 |
| hsa00061 | Fatty acid biosynthesis | 18/8016 | 0.001617035 | 0.016709357 | 0.015319275 | 55301/2180 | 2 |
| hsa04621 | NOD-like receptor signaling pathway | 181/8016 | 0.002939024 | 0.026031356 | 0.023865759 | 728358/820/1668/1669 | 4 |
| hsa04657 | IL-17 signaling pathway | 94/8016 | 0.039494086 | 0.306079164 | 0.280615871 | 3934/4318 | 2 |

Table S3 Full list of KEGG pathways of DEGs.
